# Nano-functionalization and evaluation of antimicrobial activity of *Tinospora cordifolia* against the TolB protein of *Pseudomonas aeruginosa* – An antibacterial and computational study

**DOI:** 10.3389/fmicb.2023.1138106

**Published:** 2023-04-11

**Authors:** Himporna Nath, Ankita Khataniar, Kusum K. Bania, Nobendu Mukerjee, Sami A. Al-Hussain, Magdi E. A. Zaki, Sanchaita Rajkhowa

**Affiliations:** ^1^Centre for Biotechnology and Bioinformatics, Dibrugarh University, Dibrugarh, Assam, India; ^2^Department of Chemical Sciences, Tezpur University, Tezpur, Assam, India; ^3^Department of Microbiology, West Bengal State University, West Bengal, Kolkata, India; ^4^Department of Health Sciences, Novel Global Community Educational Foundation, Hebersham, NSW, Australia; ^5^Department of Chemistry, Imam Mohammad Ibn Saud Islamic University (IMSIU), Riyadh, Saudi Arabia

**Keywords:** *Pseudomonas aeruginosa*, gold and silver nanoparticles, Cordifoliside C, TolB, Density Functional Theory, molecular docking

## Abstract

**Introduction:**

Antibacterial drug resistance, brought on by the overuse of antibiotics, is one of the biggest threats to human health. It is crucial to consider cutting-edge strategies, such as herbal remedies, to control multidrug-resistant (MDR) bacteria.

**Methods:**

This study evaluated the phytochemical, antioxidant and antibacterial properties of the various *Tinospora cordifolia* extracts. Functionalization of the isolated active compound was done using gold (Au) and silver (Ag) nanoparticles (NPs). Further, to understand the interaction of the isolated class, Cordifolisides, with its target, various in-silico methods were used.

**Results and Discussion:**

The plant was reported from the Charaideo district of Assam, whose methanolic stem extract showed the maximum activity towards the nosocomial pathogen *Pseudomonas aeruginosa*. Consequently, the active compound was isolated and characterized as belonging to the class Cordifoliside using NMR. The AuNPs and AgNPs functionalized isolates showed enhanced antimicrobial activity against *P. aeruginosa* compared to the unfunctionalized isolate. The most reactive compound, Cordifoliside C was determined using Density Functional Theory (DFT) analysis, whose interactions with the TolB protein were studied using molecular docking methods, which revealed good binding interactions of Cordifoliside C with the TolB protein.

**Conclusion:**

This study offers enormous potential for drug design and might be used as a pipeline to address the urgent problem of multidrug-resistance in bacteria.

**Figure:**
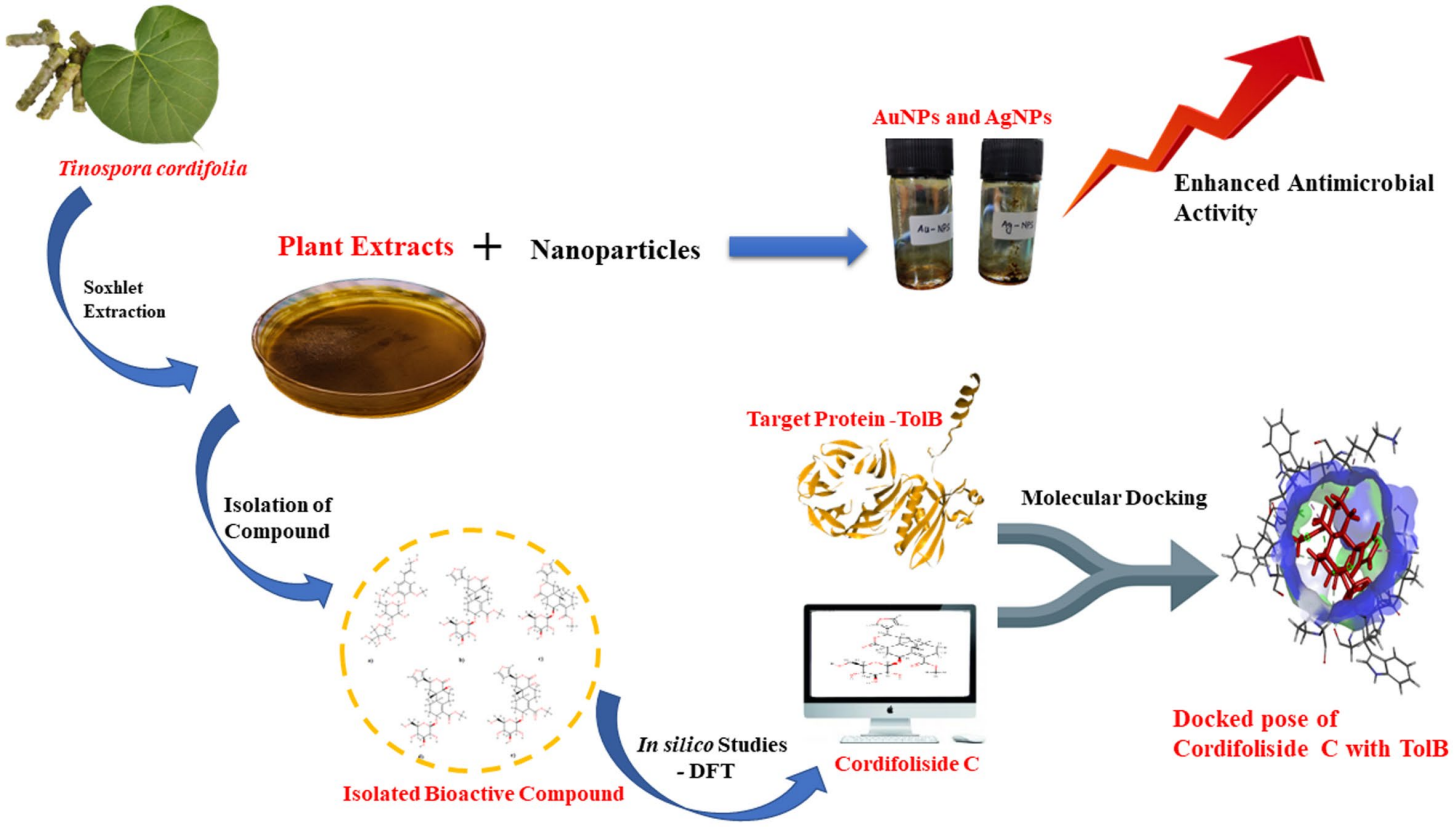
Graphical Abstract

## Introduction

Antimicrobial resistance (AMR) is a growing concern regarding human health and well-being, as ~700,000 people succumb each year to diseases caused due to AMR. The statistics are predicted to rise to 10 million deaths annually (Tagliabue and Rappuoli, 2018). Once an antibiotic’s efficacy has been proven and widely used in treatment modules, its days are counted down. Thus, the emergence of resistance is not a matter of if but of when (Davies, 1994; Levy, 1998). In a mere course of months to years, clinically significant resistance develops in pathogens such as *Enterococcus faecium*, *Staphylococcus aureus*, *Klebsiella pneumoniae*, *Acinetobacter baumannii*, *Pseudomonas aeruginosa*, and *Enterobacter* spp., grouped under the umbrella term “ESKAPE” (Davies, 1996; Rice, 2008).

The fifth most frequently isolated nosocomial pathogen is *Pseudomonas aeruginosa*, a multidrug resistance (MDR) opportunistic pathogen primarily found in medical devices such as ventilation because it prefers to grow on wet surfaces (Emori and Gaynes, 1993). It causes acute or chronic infection in immunocompromised individuals with chronic obstructive pulmonary disease (COPD), cystic fibrosis, cancer, traumas, burns, sepsis, and ventilator-associated pneumonia (VAP), including those caused by COVID-19 (Cendra Del and Torrents, 2021; Jurado-Martín et al., 2021; Rossi et al., 2021; Jangra et al., 2022). Numerous studies from India, including those by the Indian Council of Medical Research (ICMR), have highlighted *Pseudomonas* species’ resistance to monotherapy with penicillins, cephalosporins, fluoroquinolones, tetracyclines, and macrolides (Javiya et al., 2008; Ramana and Chaudhury, 2012; Rising antimicrobial resistance of Pseudomonas aeruginosa isolated from clinical specimens in India, 2013; Prakash et al., 2017; Kumari et al., 2019; Walia et al., 2019). Therefore, looking into alternative sources, such as medicinal plants, it is necessary to find new possible drug leads.

Crude extracts from plants containing a complex combination of various phytochemicals are widely used to make plant-derived medications that treat both chronic and infectious disorders (Sahoo and Manchikanti, 2013). A huge, deciduous, climbing shrub called *Tinospora cordifolia* (Willd.) Miers ex Hook. F. and Thoms, a member of the Menispermaceae family, can be found all over India, particularly in the tropical regions and ascending to an altitude of 300 m, as well as in some regions of China. Its names in Hindi and English are *Giloy* and heart-leaved moonseed plant, respectively (Saha and Ghosh, 2012). It is a constituent of multiple remedies used in India’s traditional, age-old Ayurvedic medicine to treat conditions like general sluggishness, dyspepsia, urinary infections and many others (Ahmad et al., 2009). The plant’s pharmacological activities are due to its active compounds found in various parts of the plant, including sesquiterpenoids, glycosides, steroids, diterpenoid lactones, phenolic compounds, essential oils, a combination of fatty acids and polysaccharides (Khan et al., 2017). Over 200 phytochemicals from various classes have been discovered through extensive phytochemical characterization of *Tinospora* species as reviewed (Chi et al., 2016), including diterpenoids, the most prevalent chemical class. Tinosporine, tinosporide, tinosporaside, cordifolide, cordifol, heptacosanol, clerodane furano diterpene, diterpenoid furanolactone tinosporidine, columbin, and β -sitosterol are some of the main phytoconstituents in *Tinospora cordifolia*. Its stalk has been found to contain Berberine, Palmatine, Tembertarine, Magniflorine, Choline, and Tinosporin (Jabiullah et al., 2018).

In several sectors, the creation of functionalized nanoparticles (NPs) is thought to offer the potential for usage in industries like pharmaceutical and biomedical sciences. Functionalization of the NPs is adding a chemical functional group on their surface to cover the surface of the NPs with a molecule that has the necessary chemical property with the intended application (Subbiah et al., 2010). Due to their distinct size, chemical, and physical characteristics, nanoparticles with a 1–100 nm size range are appealing multifunctional materials. The literature on NPs is pervasive, ranging from metal to polymer NPs. Among these, gold (Au), silver (Ag), silica (Si), PEG, PLGA, and PCL have received extensive study and are already used in a variety of applications, including catalysis, chemical sensing, biolabeling, photonics, and nanocarrier for drug and biomolecule delivery (Mahalingam et al., 2004; Sinha et al., 2004; Mukherjee et al., 2005; Astete and Sabliov, 2006; Targeted folic acid-PEG nanoparticles for noninvasive imaging of folate receptor by MRI – PubMed, 2008; Sharma et al., 2009). Among different nanoparticles, gold and silver are considered necessary for several applications. In the antibacterial sector, silver nanoparticles (AgNPs) are frequently employed to treat microorganisms such as fungi, viruses, and bacteria. Hybrid materials comprising Ag nanoparticles with amphiphilic hyperbranched macromolecules are created for use in surface coatings because of their demonstrated antimicrobial characteristics (Aymonier et al., 2002). Due to gold’s stability, low toxicity, large specific surface area, and ease of functionalization, nanoscale gold particles (AuNPs) have been extensively explored and used as a powerful antibacterial agent (Connor et al., 2005; Dizaj et al., 2014; Ultrastable and Biofunctionalizable Gold Nanoparticles, 2016).

Advanced drug discovery techniques using computer-based tools have emerged, allowing for screening drugs made from bioactive chemicals found in medicinal plants (Sliwoski et al., 2014). The current communication details a scientific assessment of *T. cordifolia’s* potential as an antibacterial agent and to identify the most active phytochemical present. As the nano-functionalization of plant extracts has been previously reported, the following study was conducted on the nano-functionalization of silver and gold nanoparticles on *T. cordifolia* plant extracts (Maurya et al., 2012). With the help of FCS, a secondary metabolite found in *T. cordifolia* was isolated, purified, and a study of the isolated compound’s potential antimicrobial activity with AgNPs and AuNPs was conducted. However, to the best of our knowledge, there needs to be more information available on the plant’s bioactive compounds. So, an attempt has been made to search for a target of the most reactive compound using a Density Functional Theory (DFT) and molecular docking approach.

## Materials and methods

### Experimental study

#### Chemicals and microorganisms

All the solvents/chemicals and materials used have been procured from Merck and HiMedia, India. The test organisms used in the study, *Bacillus subtilis*, *Pseudomonas aeruginosa* and *Escherichia coli*, were obtained from American Type Culture Collection (ATCC).

#### Plant material

**Figure 1:**
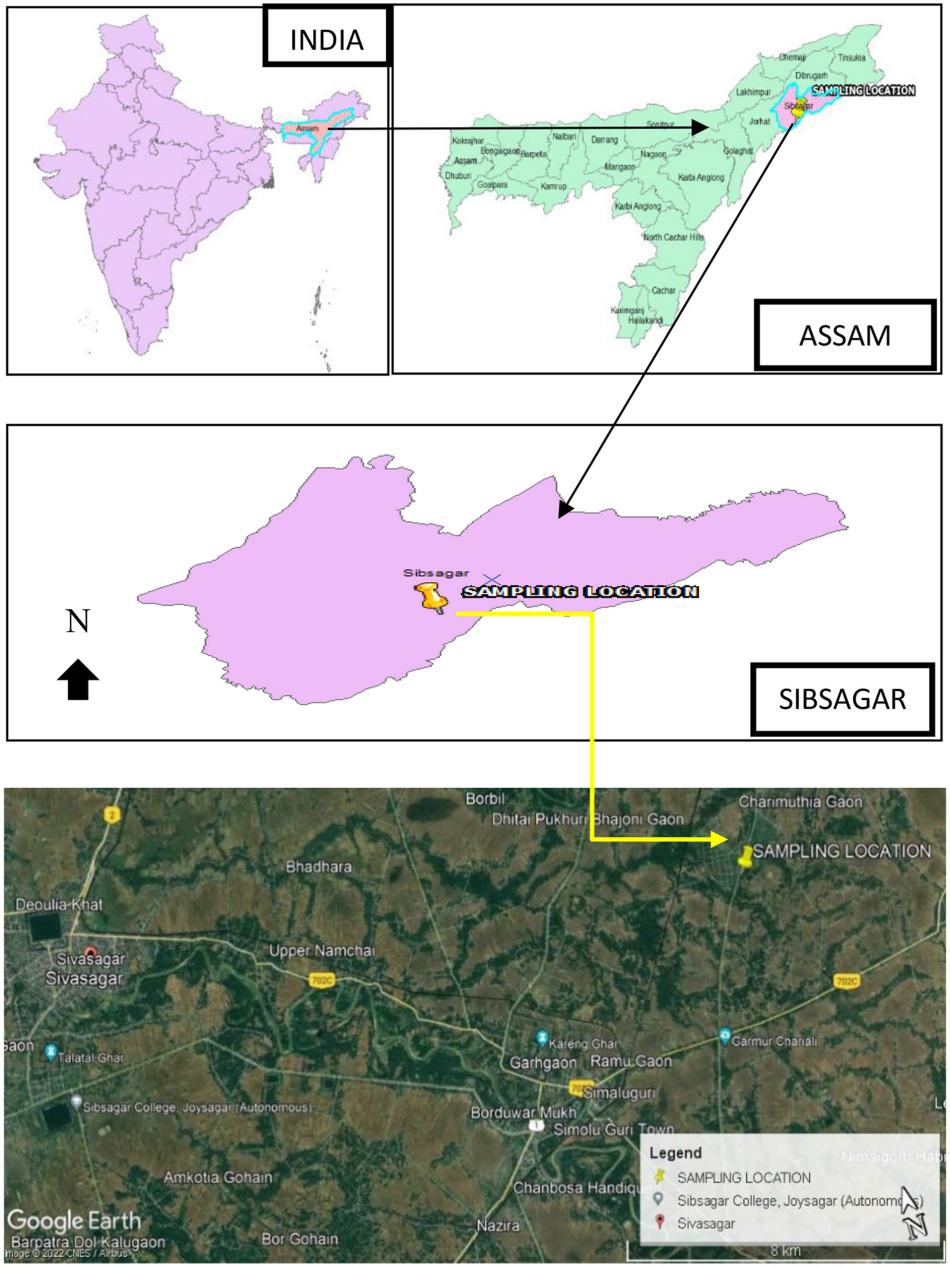
The sample collection site, Sivasagar District, India.

Fresh stems and leaves of *T. cordifolia* were collected from a kitchen garden of Silikhaguri Chetia Gaon, Sivasagar District, Assam, situated with Latitude 26.956766° and Longitude 94.806640° as shown in Figure 1. Presently the location falls under Charaideo District as it has been curved out of Sivasagar District since 2016 (Charaideo at a Glance).^1^ Plant identification was done by the Botanical Survey of India, Eastern Regional Center, Shillong.

### Plant extract preparation

**Figure 2:**
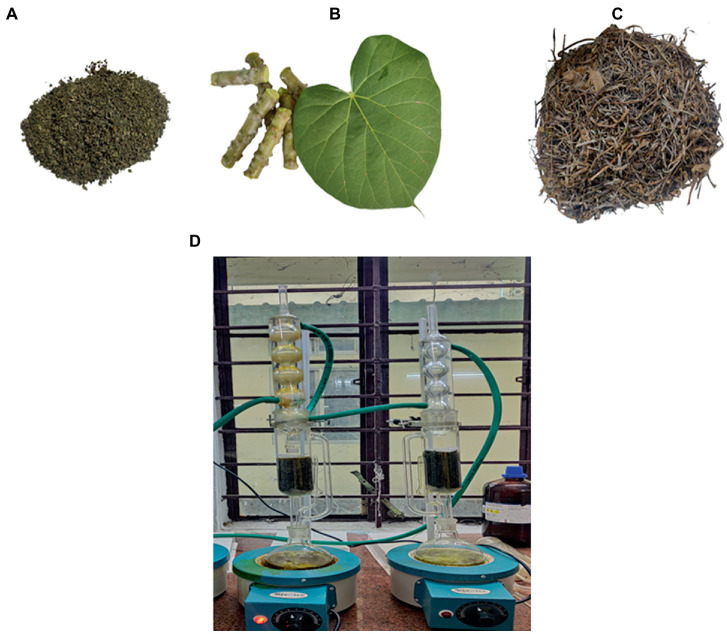
**(A)** Powdered leaves **(B)** Stems and Leaf of *Tinospora cordifolia.* **(C)** Dried stem bark before processing into powder form **(D)** Stem and leaves were extracted separately in Soxhlet extraction.

As shown in Figure 2B, the collected stem and leaves were separated and cleaned thoroughly. The plant materials were shade-dried (Figures 2A,C), and the dried leaves and stems were crushed and coarsely powdered by using a mechanical grinder. The powdered stem and leaf material were extracted separately using a Soxhlet extractor, as shown in Figure 2D (Prasad and Chauhan, 2019).

The plant material was extracted with 2.5 L of hexane, chloroform, methanol, and water using a Soxhlet continuous extraction system. Coarse powder of about 30 g (both for stem and leaf) was introduced into the extractor separately and extracted with hexane in a Soxhlet extractor. The crude extract was shade dried and further extracted with chloroform, followed by methanol and water. The plant materials were extracted until the solvent ran clear. The extracts were filtered with Whatman filter paper no. 1. The resulting extracts were concentrated and dried using a rotary evaporator and stored at 4°C (Arora et al., 2012; Rana and Suttee, 2012; Suttee et al., 2013; Kaur G. et al., 2016; Kaur H. et al., 2016; Ahmed, 2019). The extraction yield was calculated using the following formula (Shrestha and Lamichhane, 2021):


$$Extraction\;yield\;(\%)\;=\;\;\frac{weightoffinalextractobtained\;}{totalweightofsampleused}\times 100$$

From 30 g of powdered plant material, the weight of each solvent extract and their percentage yield after extraction are tabulated below (Table 1).

**Table 1:** Physical characteristics, weight of solvent and the percentage yield of plant material.

S. no.	Solvent	Extract	Appearance	Consistency	Weight (in g)	Yield %
1	Hexane	Leaves	Dark brown	Sticky solid	3.067	10.2
		Stem	Dark brown	Sticky solid	2.102	7
2	Chloroform	Leaves	Light brown	Sticky solid	5.724	19
		Stem	Dark brown	Semi-solid	2	6.7
3	Methanol	Leaves	Dark brown	Viscous	6.142	20.4
		Stem	Dark brown	Viscous	3.07	10.2
4	Aqueous	Leaves	Dark brown	Viscous	12.91	43
		Stem	Dark brown	Viscous	10.32	34.3

Highest percentage of yield was found in aqueous extract of leaf (43% w/w), followed by aqueous stem extract (34.3% w/w) while the lowest in chloroform extract of stem (6.7% w/w). This solvent extract was further used for various purposes.

### Preliminary phytochemical screening

The phytochemical profile of *T. cordifolia* extracts was determined through phytochemical screening tests to determine the relative abundance of secondary metabolites. Standard protocols were used to conduct several tests, including the Alkaline detection assay (flavonoids), Dragendroff’s assay (alkaloids), Millon’s reagent test (amino acid), Ferric chloride test (phenols), Gelatin test (tannins), Fehling’s test (carbohydrates), Legal Test (glycosides) and Froth formation test for saponins (Auwal et al., 2014).

### Antioxidant activity-DPPH radical scavenging assay

Based on the scavenging activity of the stable 1,1-diphenyl-2-picrylhydrazyl (DPPH), the antioxidant activity of the extracts from Giloy leaves and stems was evaluated with some minor modifications. Concentrations of plant extracts (100, 200, 300, 400, 500, 600, 700, 800, 900, and 1,000 μg/ml) were prepared. Quercetin was used as a standard for this assay. Different volumes (100–1,000 μg/ml) of plant extracts were made up to 2 ml with methanol, and 1 ml DPPH (0.1 mM) solution was added. The reaction mixture was incubated in dark conditions at room temperature for 30 min. After 30 min, the absorbance of the mixture was read at 517 nm against a blank. The test was done in triplicates (Upadhyay et al., 2014).

The % radical scavenging activity of the plant extracts was calculated using the following formula,


$$\mathrm{Inhibition}\%=\frac{A_{0}-A_{1}}{A_{0}}\times 100$$

where, A_0_ = Absorbance of control; A_1=_ sample absorbance.

### Antimicrobial assay

Antimicrobial activity of all four extracts (hexane, chloroform, methanol and aqueous) was checked against three pathogenic bacterial strains. Gram-positive bacterial strains, such as *Bacillus subtilis* (ATCC 6051) and gram-negative bacterial strains, *Pseudomonas aeruginosa* (ATCC 27853) and *Escherichia coli* (ATCC 25422) were used. Freshly grown bacterial inoculum for each strain was mixed with normal saline to adjust the turbidity to 0.5 McFarland standards giving a final inoculum of 1.5 × 10^8^ CFU/ml (Antimicrobial Susceptibility Testing, 2005).

Two different methods were employed; the antibacterial activity of the four crude extracts was determined using the agar well-diffusion method, while the nanofabricated extracts were determined using the disc diffusion method.

Mueller Hinton Agar (MHA) petri plates were inoculated with 50 μl of bacterial suspensions for the agar well diffusion method. A well was bored at the center of the plate of uniform diameter 6 mm with a sterile aluminum borer, and 50 μl of each extract was added to the wells and incubated for 24 h at 37°C. After incubation, the plates were checked for the zone of inhibition (measured in mm). The activity of the extracts was compared with standard antibiotic Ciprofloxacin as positive control and DMSO as a negative control. Initial antimicrobial screening revealed the most potent extract, which was further used to determine the structure of the compound. A comparative antibacterial test was done using the disc diffusion method between the nanofabricated extracts and the compound isolated from the most potent extract to see if the antimicrobial activity was enhanced.

The broth microdilution method was used to measure the MICs of the extract showing the highest zone of inhibition, the isolated compound, and the nanoparticles. The protocol used adhered to CLSI recommendations (VET04Ed3, 2014; VET03Ed2, 2020). Mueller-Hinton broth was used to create the serial dilutions from the stock solution in 96-well microplates, ranging from 32 mg/ml to 0.25 mg/ml. 100 μl of 5 × 105 CFU/ml bacterial suspension was added to each well, after which 50 μl different concentrations of extracts were added to the wells. The microtiter plates were incubated for 24 h at 37°C. The minimum inhibitory concentration was thus determined to be the lowest concentration of each extract that showed no visible growth (Mogana et al., 2020).

After 24 h of incubation at 37°C, the minimum bactericidal concentration (MBC) was determined to be the lowest extract concentration that killed 99.9% of the bacterial inocula. The Ozturk & Ercisli technique was used to determine MBC (Chemical Composition and in vitro Antibacterial Activity of Seseli libanotis, 2006). All extracts, nanoparticles, and the isolated compound were subjected to MBC. Ten microliters were collected from the wells of the MIC microtitre plate and spread on MHA plates and two wells above the MIC value well. After 18–24 h of incubation at 37°C, the colony count was done. The MBC value was determined to be the sample concentration that generates fewer than 10 colonies (Mogana et al., 2020).

### Compound isolation and NMR

Fractions from the sample extract were isolated using flash chromatography. Silica gel 60/120 mesh infused with 100% chloroform was used to pack a 5-inch column. The crude extract was dissolved in 100% chloroform along with silica. After the solvent was evaporated, the silica, infused with the extract, was added to the column. A gradient solvent system of a ratio of Chloroform and Methanol (100% chloroform, 9:1, 8:2 to 100% methanol) was used. A total of 20 fractions were collected in test tubes and ran directly on TLC plates. Fractions having the same R_F_ (retardation factor) values were pooled and evaporated on a rotatory evaporator (Flash Chromatography, 2016). The sub-fractions were used to test antimicrobial activity against *Pseudomonas aeruginosa*. The sub-fraction, which showed bioactivity, was dried using a rotary evaporator to yield the isolate. The yield percentage was calculated using the following equation.


$$Yield\%=Weightofisolate\times \frac{100}{Weightof\;dry\;plantmaterial}$$

The yield percentage of the isolate was found to be 8.5%. The isolate was then characterized by NMR. The ^1^H and ^13^C nuclear magnetic resonance (NMR) analyses were done by DRX-400 Varian, Bruker AVANCE III HD 400 MHz spectrometers. Chemical shifts (δ) are reported in ppm downfield from tetramethylsilane.

### Preparation of silver and gold nanoparticles

100 μl of Au and Ag solutions were added to 100 mg of isolated plant extracts to get the Au and Ag-loaded material. The 0.01 M strength of the Au solution was prepared by dissolving gold (III) chloride (AuCl_3_) in water, and 1–2 drops of HCl were added to digest the Au completely. To the Au solution, L-valine amino acid was added to stabilize the Au and sodium borohydride (NaBH_4_) was used to reduce the gold. A similar method was used to prepare an Ag solution in which silver nitrate (AgNO_3_) was used as the source of Ag. The molecules have functional groups such as -OH and ketones. The nanoparticles will interact through these functional groups. Thus, the silver nanoparticles (AgNPs), gold nanoparticles (AuNPs), and the isolated compound were now subjected to antimicrobial activity analysis. Scanning electron microscopy (SEM) images and an EDX spectrum were used to determine the nanoparticles’ presence, size, shape, and dispersion (Im et al., 2012). Energy dispersive X-ray (EDX) analysis was performed with JEOL, model No: 7582 (Oxford make), resolution: 137 eV at 5.9 KeV; Minimum weight % = 0.01%, sample size: 10 mm dia, 1 mm thick(max), dry and moisture free and Scanning electron microscopic (SEM) images were recorded with JEOL, JAPAN (Model: JSM 6390LV) with resolution: 3 nm, magnification: 3,00,000×, applied voltage: 30 kV (max.).

### Antimicrobial evaluation of AgNPs, AuNPs, and Cordifolisides

Disc diffusion method was used to test the antibacterial activity of AgNPs, AuNPs, and well diffusion for the isolated compound. MHA plates were inoculated with 50 μl of *P. aeruginosa* culture. Sterile discs (6 mm), impregnated with the nanoparticles solution (50 μl), were placed on the top of MHA agar plates and incubated at 37°C for 24 h. On the other hand, agar well diffusion was used to determine the isolated compound’s bacterial susceptibility. Zone of inhibition for AuNPs, AgNPs and Cordifolisides were measured and recorded (Vanaja and Annadurai, 2013).

### Computational approach

#### Selection of compounds using DFT

**Figure 3:**
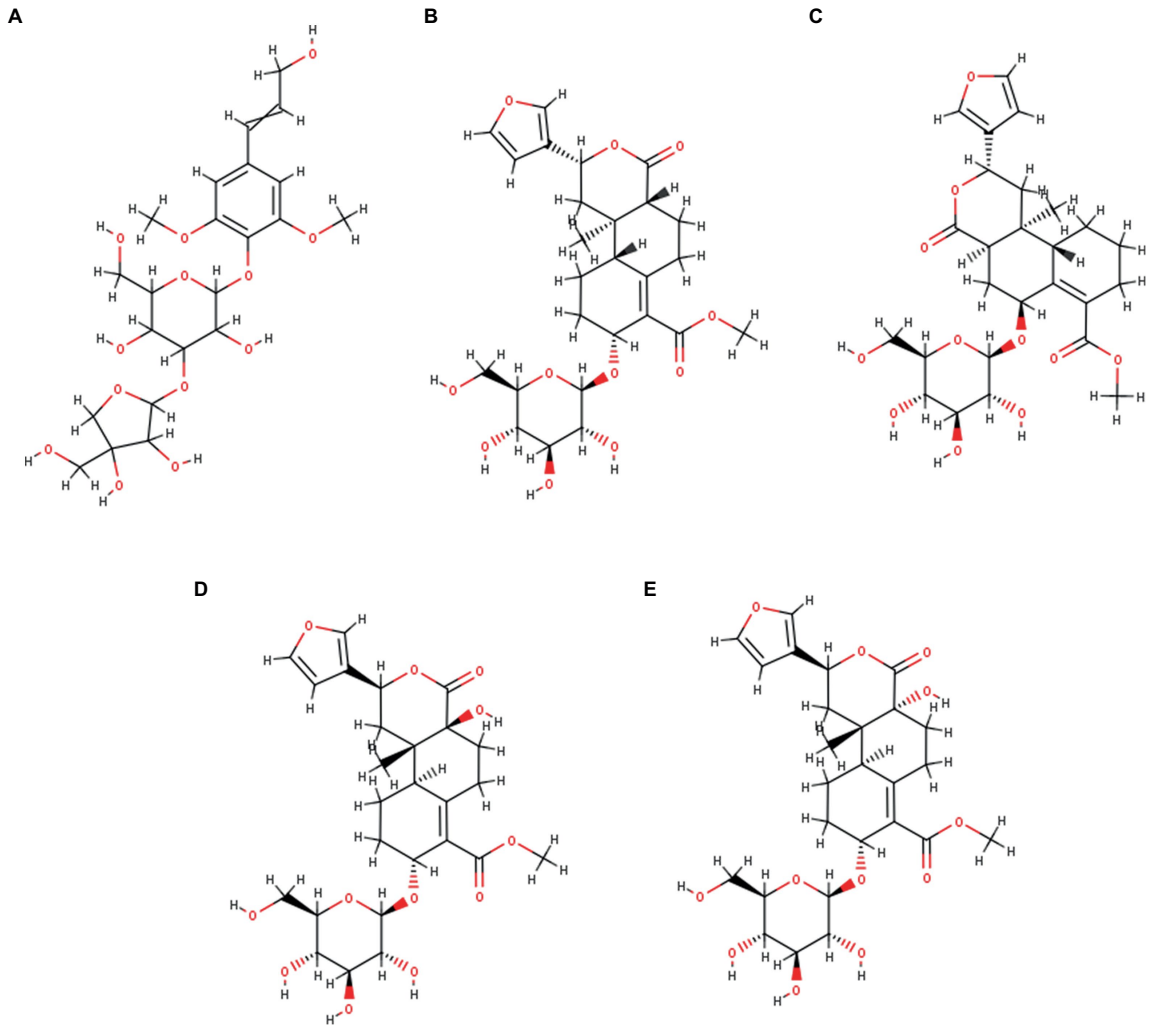
Five Cordifoliside compounds, **(A)** Cordifoliside A, **(B)** Cordifoliside B, **(C)** Cordifoliside C, **(D)** Cordifoliside D, **(E)** Cordifoliside E.

A comprehensive review of the scientific literature revealed that *T. cordifolia* has a wide range of phytochemicals. Among them, a set of norditerpene furan glycosides, Cordifolisides, were selected for our study based on the structure determined from the most active *T. cordifolia* extract. The structures of Cordifoliside A, B, C, D, and E used in our study are shown in Figure 3.

Complete geometry optimization of all five molecules in the gas phase was carried out at gradient-corrected DFT using DMol^3^ incorporated in BIOVIA Discovery Studio v4.5 (DS v4.5) (BIOVIA discovery studio – BIOVIA – Dassault Systèmes®, n.d.). The descriptors were calculated and analyzed using an approach that was previously reported in a work (Rajkhowa et al., 2022b).

#### Prediction of potential target

Out of the four plant extracts, preliminary antimicrobial screening allowed us to select one extract with the highest zone of inhibition. As Cordifolisides and nanofabricated extracts showed enhanced antimicrobial activity toward *Pseudomonas aeruginosa*, we also tried to understand its mechanism of action against the TolB protein, which is essential for *P. aeruginosa* growth. Almost all Gram-negative bacteria have a multi-protein complex called the Tol-Pal system, which connects the cytoplasmic (or inner) membrane with the outer membrane. TolB is the periplasmic component of the Tol-Pal system. TolB is one of the most prevalent proteins in the periplasm of the human pathogen *Pseudomonas aeruginosa*, according to a proteomic investigation. Numerous attempts to produce TolB mutants in this bacterium using site-directed or extensive random transposon mutagenesis failed, indicating that TolB may be necessary for *P. aeruginosa* survival (Sciuto et al., 2014). TolB is a soluble protein that resides in the periplasmic space (Lazzaroni et al., 2002; Imperi et al., 2009), making it more accessible to drugs than cytosolic targets because here, drugs only need to permeate the outer membrane. Thus, the accessibility and essentiality of periplasmic proteins for survival and growth makes them good choice of targets. Since there is lesser information available about the target proteins of phytochemicals of the plant, so in our *in-silico* study, an attempt has been made to check the binding interactions of TolB as a target protein with the most active compound identified from the plant using Density Functional Theory (DFT).

#### Molecular docking studies

In addition to a thorough assessment of the literature, binding and active sites in proteins are frequently connected to the protein’s structural cavities and pockets. In this investigation, the active site was identified using the Discovery Studio(DS v4.5) and a thorough literature review (Bouveret et al., 1995). To study the interaction between a protein and a bioactive substance, docking simulation was performed using the CHARMM-based docking software (CDOCKER) of the DS v4.5. While non-bonded interactions were eased during the docking simulation by utilizing this algorithm, ligands were kept flexible. In reported literature, the CDOCKER algorithm that we employed is described in depth (The CHARMM22 Force Field, 2003). The protein structure was kept rigid during the whole docking process (Wu et al., 2003).

A molecular docking approach was used with the target protein TolB/Pal complex (PDB ID: 2HQS), downloaded from the database,^2^ in order to evaluate the binding affinity of our compound. The Tol-Pal system is comprised of five core proteins; the inner membrane (IM) proteins TolA, TolQ, and TolR communicate with one another through their transmembrane helices, while TolB and Pal form a complex at the outer membrane (OM) and interact with the periplasm-spanning TolA (Lloubès et al., 2001; Lazzaroni et al., 2002). The Pal region has been removed for this study, and the structure has been cleaned and optimized using the protein preparation module of DS v4.5. The protein model was optimized using a CHARMM-based force field (steepest descent method followed by a conjugate gradient) in default mode. The active site of the protein was manually predicted and identified using the “edit binding site” module of DS v4.5. The Discovery Studio Visualizer was used to further research the docked structure with the lowest energy.

## Results

### Preliminary phytochemical screening

The results for the different types of phytochemicals’ presence are shown in Table 2. In this study, a variety of solvents, including methanol, hexane, aqueous, and chloroform, were used. The findings of this study demonstrated that various solvents influenced the phytochemical components included in the extracts. Leaf extract indicated the presence of maximum compounds, while methanolic extracts revealed the highest bioactive compounds.

**Table 2:** Phytochemical screening of various extracts of *T. cordifolia*.

S. no.	Chemical constituents	Tests	Hexane extract		CHCl₃ extract		MeOH extract		Aqueous extract	
			Stem	Leaf	Stem	Leaf	Stem	Leaf	Stem	Leaf
1	Alkaloids	Dragendroff’s Test	+	+	+	+	+	+	−	+
2	Carbohydrates	Fehling’s Test	−	+	+	+	+	+	−	+
3	Proteins and Amino acids	Milon’s Test	−	−	−	−	=−	=+	−	+
		Ninhydrin test	−	−	−	−	−	+	−	+
4	Flavonoids	Alkaline reagent test	+	+	+	+	+	+	+	+
5	Tannins	Gelatin test	−	−	−	−	−	+	−	+
6	Steroids and Tritrpenoids	Salkowski Test	−	+	−	−	−	+	−	−
7	Glycosides	Legal Test	−	+	−	+	+	+	+	−
8	Saponins	Forth formation test	−	+	−	+	+	+	−	+

(−) A sign indicates the absence of a constituent in the respective screening test; (+) sign indicates the presence of a constituent in the respective screening test.

### Evaluation of antioxidant activity-DPPH radical scavenging assay

**Figure 4:**
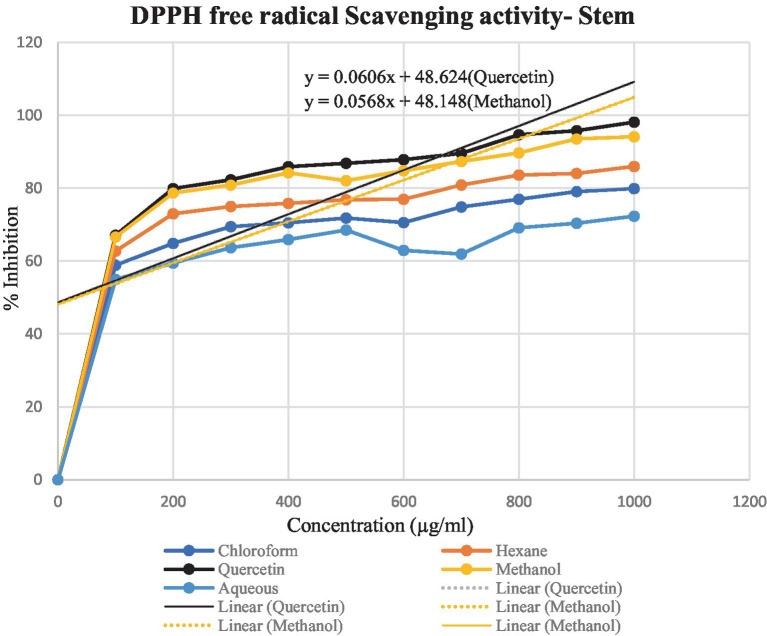
Graphical representation of the percentage of inhibition of hexane, chloroform, methanol and aqueous extracts for *T. cordifolia* stem.

**Figure 5:**
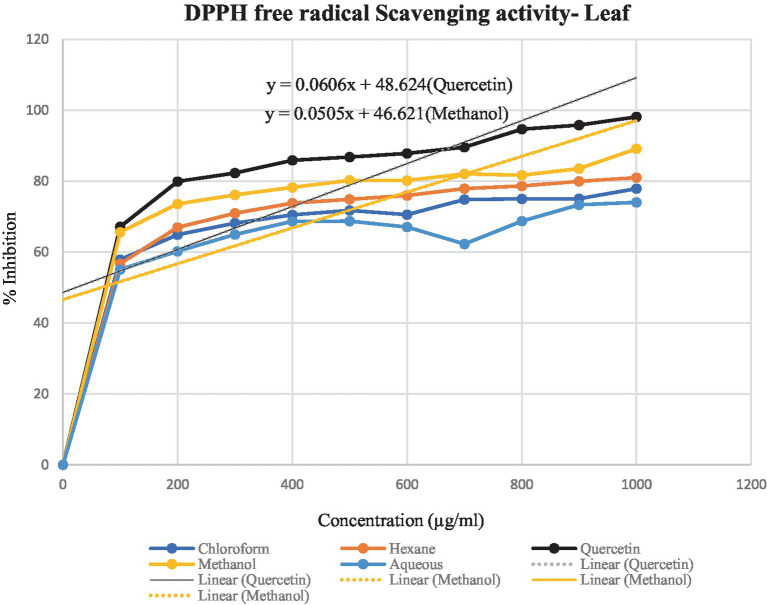
Graphical representation of *T. cordifolia* leaf for the percentage of inhibition of various extracts.

DPPH assay was used to calculate the percentage of inhibition for the given extracts – hexane, chloroform, methanol and aqueous. In Figures 4, 5, the percentage of inhibition of stem and leaf extracts are shown in graphical data, respectively. It is evident from both figures that the scavenging activity of methanolic stem extract of *T. cordifolia* is comparable to the standard Quercetin. The present study revealed that methanol was the better extractive solvent for antioxidant activity. The result coincides with the view of a reported literature (Sivakumar, 2010).

### Antimicrobial activity of *Tinospora cordifolia* extracts

The results of the antimicrobial screening assays are presented in Tables 3, 4.

**Table 3:** Zone of inhibition (Leaf extract).

Microorganism	Concentration of extract (μg/ml)	Control	Hexane extract	CHCl₃ extract	MeOH extract	Aqueous extract
		Zone of Inhibition (mm)				
*E. coli*	50	38 ± 0.03	19 ± 0.01	13 ± 0.09	20 ± 0.04	Insignificant
*P. aeruginosa*	50	41 ± 0.06	25 ± 0.04	22 ± 0.02	28 ± 0.07	Insignificant
*B.subtilis*	50	35 ± 0.02	26 ± 0.03	23 ± 0.07	27 ± 0.04	Insignificant

**Table 4:** Zone of inhibition (Stem extract).

Microorganism	Concentration of extract (μg/ml)	Control	Hexane extract	CHCl₃ extract	MeOH extract	Aqueous extract
		Zone of Inhibition (mm)				
*E. coli*	50	38 ± 0.03	20 ± 0.07	28 ± 0.01	29 ± 0.01	Insignificant
*P. aeruginosa*	50	41 ± 0.04	8 ± 0.03	18 ± 0.04	**31 ± 0.09**	Insignificant
*B.subtilis*	50	35 ± 0.04	28 ± 0.06	21 ± 0.06	29 ± 0.05	Insignificant

For leaf extract: Table 3.

For stem extract: Table 4.

The four solvent extracts of leaves and stem of the plant were tested for antibacterial activity, respectively, by the agar well diffusion method. Methanolic crude extract of *Tinospora cordifolia* outperformed the other three solvents in terms of activity against the test microorganisms. In the case of *P. aeruginosa*, *T. cordifolia* methanolic stem extract has been showing excellent zone of inhibition which was comparable to that of Ciprofloxacin. Meanwhile, the aqueous extract showed very marginal zone of inhibition that could not be measured in accurate measurement. Keeping in view of the highest zone of inhibition of methanolic stem extract, it was further taken for compound extraction and nano-formulation.

### Compound isolation and NMR

**Figure 6:**
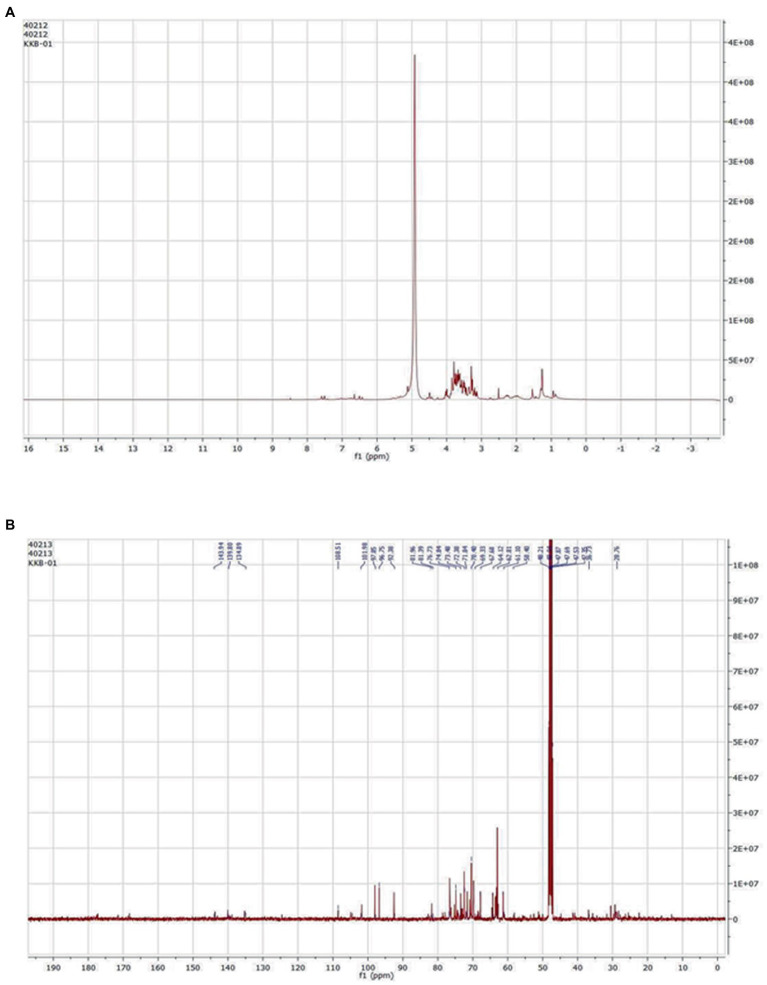
**(A)** ^1^H and **(B)** ^13^C NMR.

Among the 20 fractions collected from flash chromatography, five fractions (Fraction 16–20) showed a single spot on TLC with the same R_f_ value. Samples with nearly equal R_f_ values were pooled into one fraction (Fraction A) based on the TLC results. Fraction A was then evaporated to remove the eluent contained therein, thus isolating the pure compound from it. The ^1^H and ^13^C NMR spectrum of the compound resembled with the NMR pattern of Cordifoliside class of *Tinospora cordifolia* as shown in Figure 6. The chemical shift values (δ) were compared with the literature data (Gangan et al., 1994) and were found to be matching with the reported values. In the ^1^H NMR spectra, methyl proton (CH_3_) are appeared in the 0.75–1.25 ppm in aliphatic region (-CH_2_) proton of cyclohexane and methylene protons are appeared in 1.50–3.00 ppm range. Methoxy proton (-OCH_3_) is in the range 3.6–3.9 ppm and the (CH) proton of tetrahydropyran are in the range of 3.60 3.99 ppm. The alcoholic proton (-OH) are in the range of 4.00–4.50 ppm. Furan (CH) proton are appeared in the aromatic region 6.40–7.50 ppm range. In the ^13^C –NMR spectra CH_3_ Carbon is appeared at 16.0 ppm. Aliphatic and cyclohexane (CH_2_) carbon are appeared in the 22.0–45.0 ppm range. Methoxy carbon (-OCH_3_) and aliphatic (CH_2_) carbon are appeared in the 55.0–60.0 ppm range. Tetrahydropyran (CH) and cyclohexane carbon are appeared in 65.0–101.0 ppm range. Furan carbons are appeared 108-143 ppm range. Carbonyl carbon(C=O) appeared in 165–172.0 ppm range. Based on the NMR analysis, it was thus, confirmed that the plant extract that was highly polar in nature and constituted of the Cordifoliside family. The expected molecules are shown in Figure 3, the dataset can be found in the online repository, Figshare (NMR.rar, 2023).

### Characterization of Au nanoparticles

**Figure 7:**
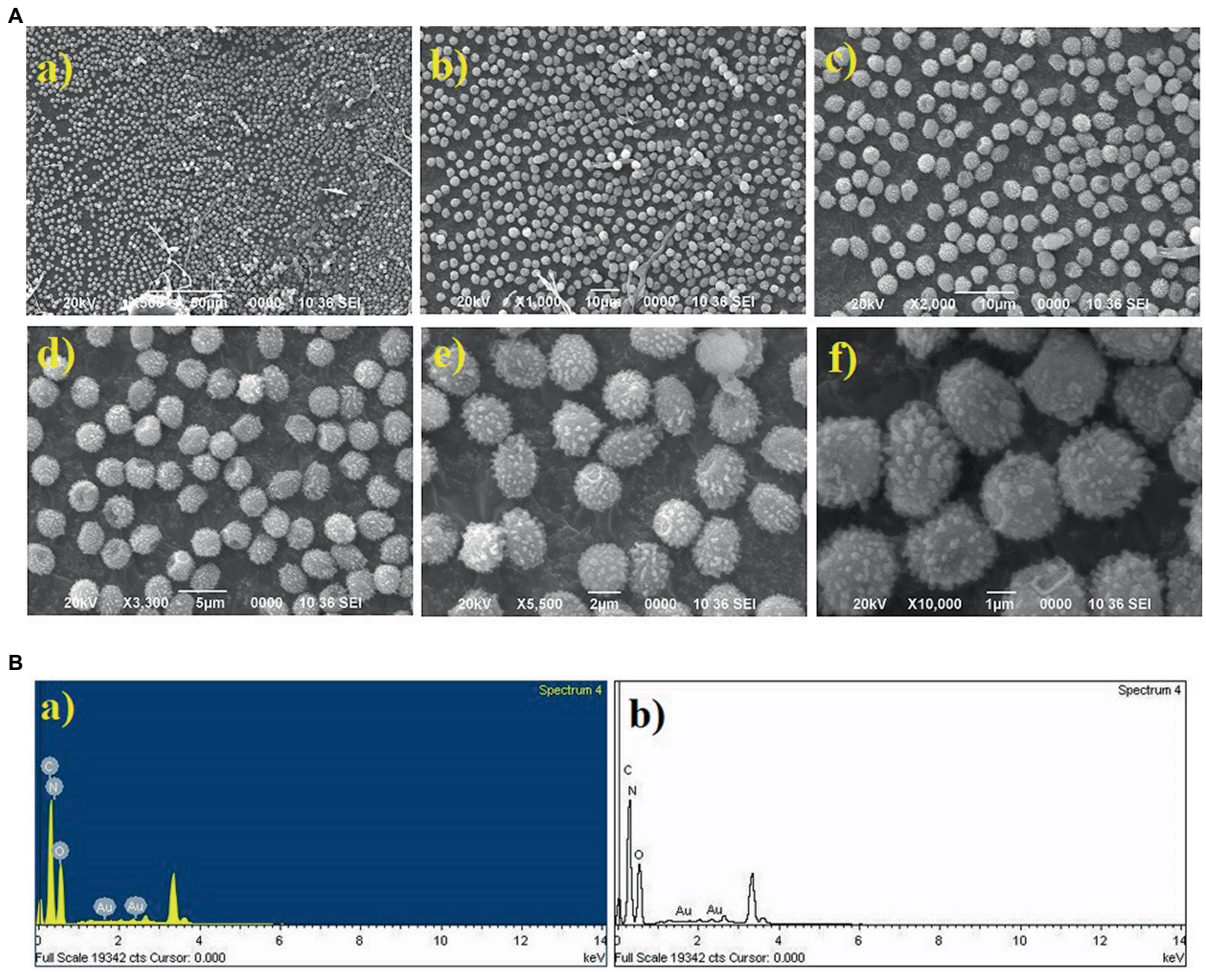
**(A)** SEM image of Au loaded compound. **(B)** The EDX spectrum of the material.

The gold (Au) and silver (Ag) functionalized compounds were characterized by SEM and EDX analysis. The SEM image of Au loaded compound is shown in Figure 7A. The SEM image of Au loaded compound represented a beautiful spherical Au-nano structure. The spherical gold particles were found to be highly distributed on the surface of the compound. The SEM image also indicated for the metal-to-surface interaction that may impart or enhance the biological activity of the compound. The presence of Au was identified through the EDX analysis. The EDX spectrum of the material is shown below in Figure 7B showing the presence of Au.

### Characterization of Ag nanoparticles

**Figure 8:**
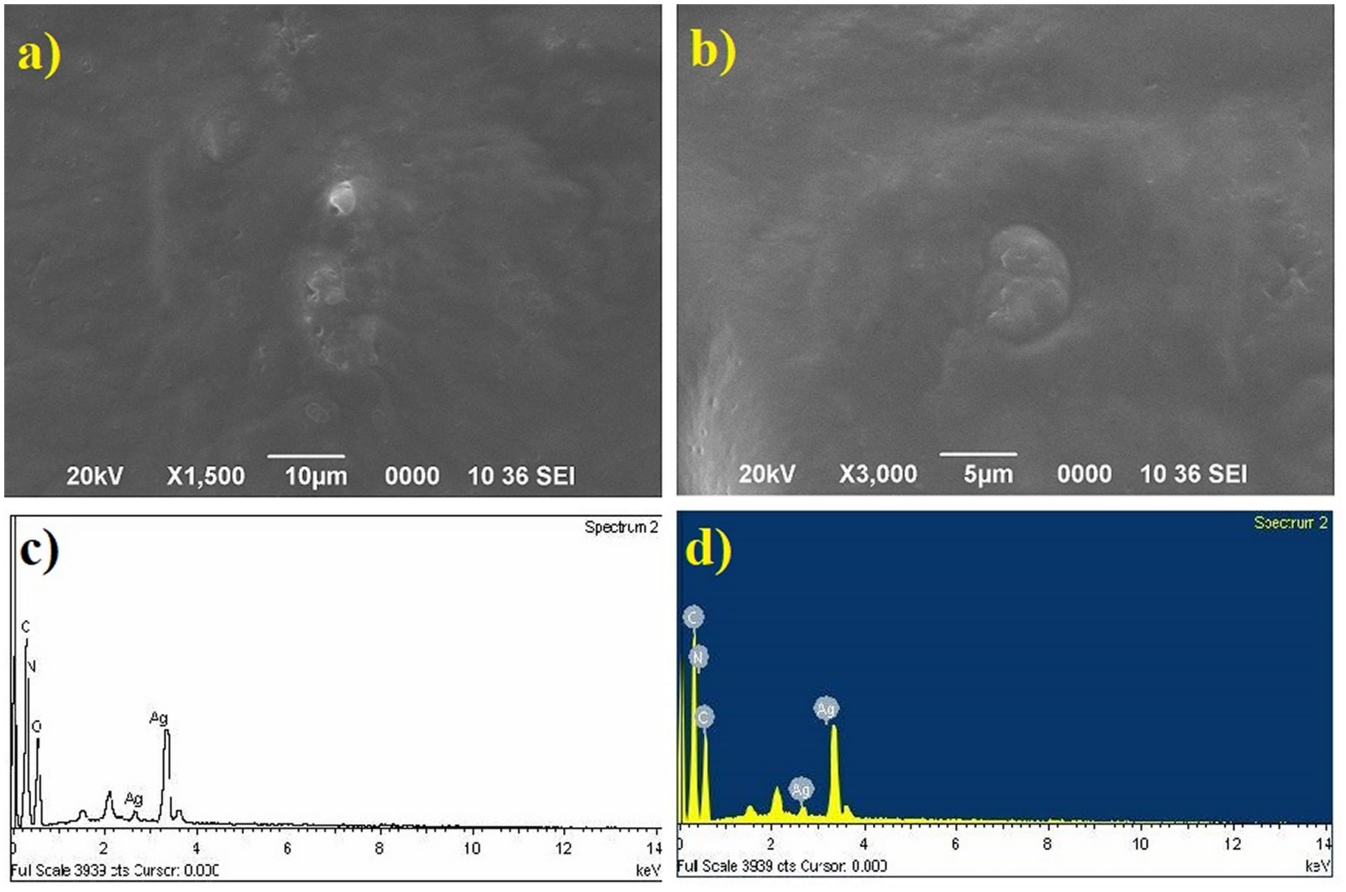
SEM image of Ag loaded compound and the EDX spectrum.

SEM image of Ag loaded compound was however found to be completely different from those of the Au loaded compound. Like the Au particles, the Ag particles were not found to be located on the surface of the compound. The absence of any surface-bound Ag material implied that the Ag particles were probably diffused into the surface matrix. Since the Ag particles are more polar in comparison Au, so the electrostatic force of interaction existing between the Ag and the organic matrix probably favored the high diffusion of Ag into the interior part of the material. This strong interaction and high diffusion of Ag lead to the high activity of the material in comparison to the one that is modified with Au. The presence of Ag was also confirmed from the EDX analysis and the EDX spectrum is spectrum is shown below in Figure 8.

**Figure 9:**
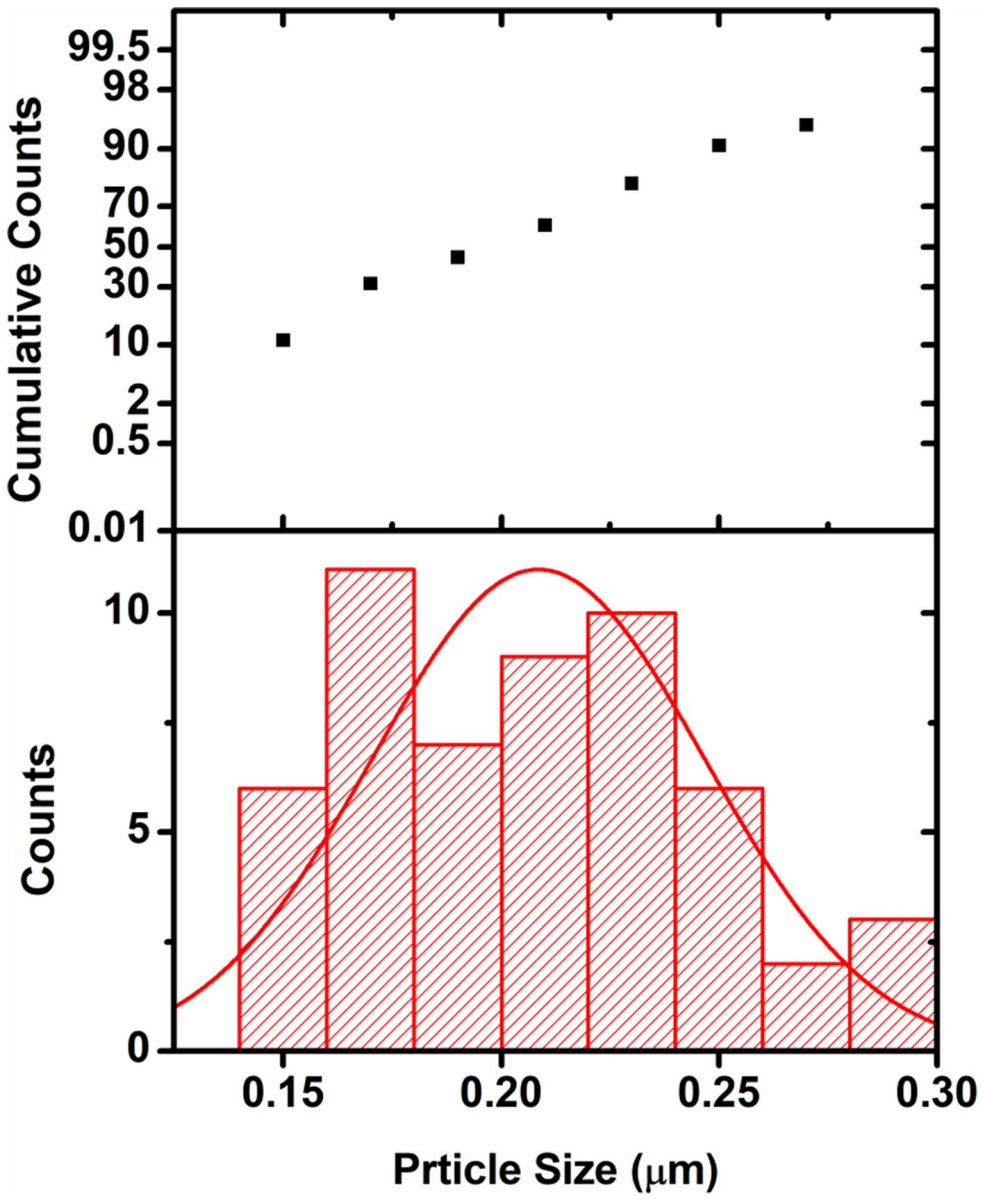
Graph showing the particle size distribution of Au-particles. The analysis was done considering the SEM-image Figure 7A(f).

The particle size distribution analysis was also performed from the SEM images. From the analysis it was found that the Au-particles were in the range of 0.15–0.30 μm, Figure 9. As the Ag-particles mostly got diffused into the matrix so their particles were not clearly seen at the external surface. Probably, Ag-particles formed in the nanometer (nm) dimension and hence easily got diffused into the matrix while most of Au-particles got agglomerated into larger dimension and remained intact at the external surface and hence its activity was found to be less than of the one modified with Ag. It is pertinent to be mentioned herein that the particle size analysis was done based on SEM images. Due to certain limitations we could not performed the TEM analysis which would have provided us the clear picture about the particles size. The present analysis however provided us the information that Au-particles due to their agglomeration into micrometer (μm) dimension could not diffuse into matrix and hence could not bring high activity like that of Ag.

### Antimicrobial activity of Cordifolisides, AgNPs, and AuNPs extracts

In the past, silver compounds and silver ions were known for their inhibitory effects and used as effective therapeutic agents for avoiding wound infections. The strong interaction between silver and the thiol groups found in bacterial respiratory enzymes is what causes silver to have an inhibiting effect on bacterial cells (Gordon et al., 2010). Rajesh Kumar in his study suggested that AgNPs possess impressive antibacterial action against pathogenic organisms that are antibiotic-resistant. The antibacterial activity of antibiotics was improved by AgNPs. Due to prolonged exposure, bacteria developed resistance to antibiotic treatment. The results of his study indicate that AgNPs have the ability to increase the antibacterial activity of antibiotics and have potential applications in the medical field (Rajeshkumar, 2017). In our experiment, when we compared the antibacterial activity of AgNPs, AuNPs and Cordifolisides, it was found that AgNPs have shown more antibacterial activity than the other extracts (Table 5).

**Table 5:** Enhanced antibacterial activity of AgNPs and AuNPs against pathogenic bacteria *P. aeruginosa.*

Microorganism	Concentration of extract (μg/ml)	Control	Cordifolisides	AgNPs	AuNPs
		Zone of Inhibition (in mm)			
*P. aeruginosa*	50	41 ± 0.04	22 ± 0.01	**31 ± 0.06**	24 ± 0.03

**Figure 10:**
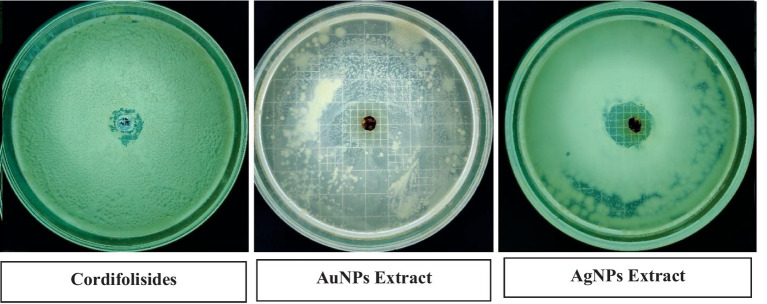
Nanoparticles coated discs had increased antibacterial activity against *P. aeruginosa.*

The enhanced antibacterial effect was highly observed against *P. aeruginosa* (Figure 10). This result clearly shows that antibiotic-resistant bacteria, *P. aeruginosa* was sensitive to the AgNPs and AuNPs impregnated discs.

**Figure 11:**
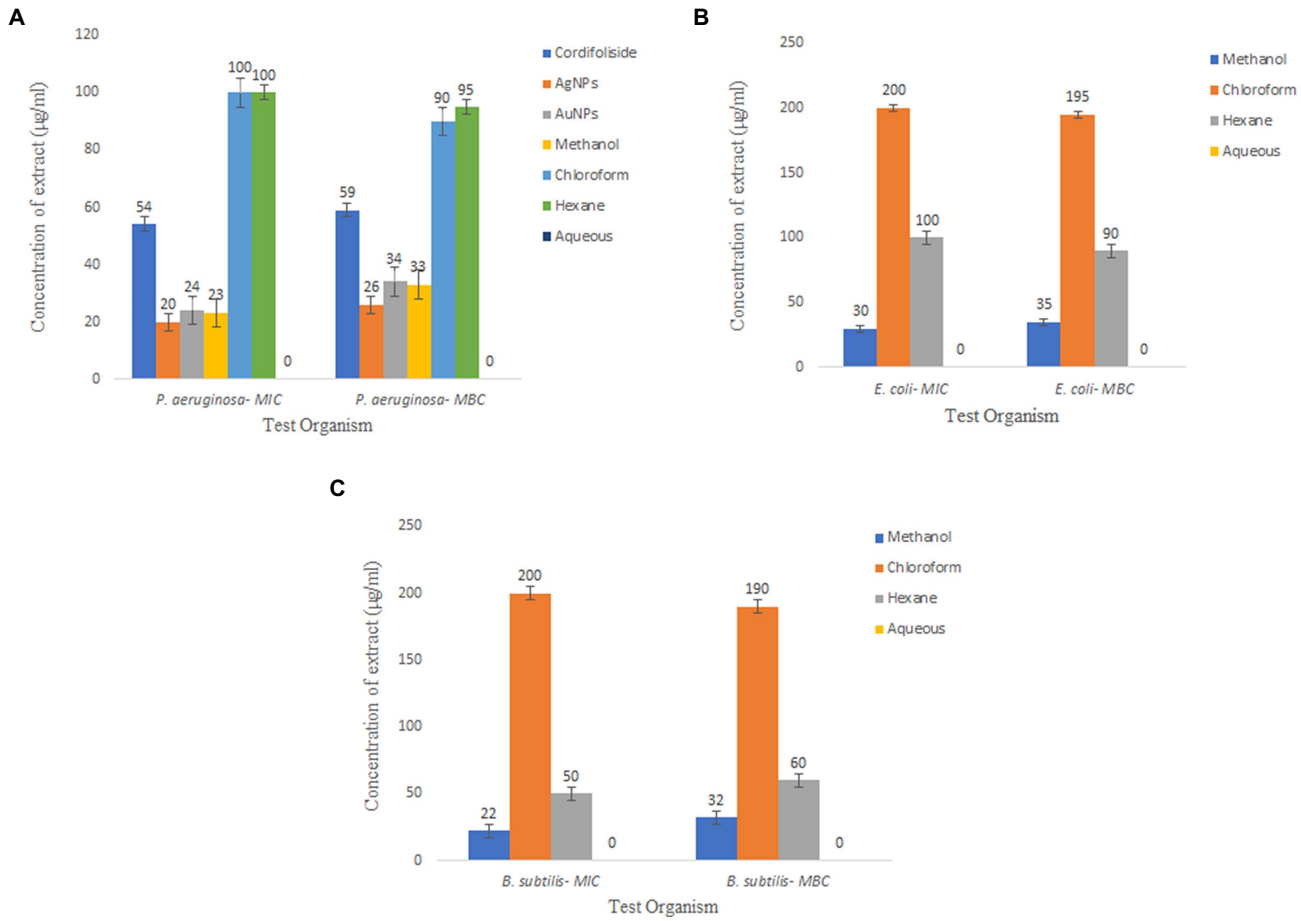
Minimum Inhibitory Concentration and Minimum Bactericidal Concentration of *T. cordifolia* extracts tested against **(A)** *P. aeruginosa,* **(B)** *E. coli,* **(C)** *B. subtilis.*

As determined by the broth dilution technique, the minimum inhibitory concentration of the extracts against different bacteria is depicted in Figure 11. The extracts are more active against the test microorganism when the MIC is smaller (13.5A: Minimal inhibitory concentration (MIC), 2018). In this study, for *P. aeruginosa* the best MIC value was observed in AgNPS, methanolic stem extract and AuNPs.

### *In silico* screening of phytochemicals using density functional theory

For determining functions like electron affinities, ionization potentials, orbital energies, and molecular structures, DFT analysis is of utmost importance (Rajkhowa and Deka, 2014). A thorough prediction of the pharmacological activities of the phytochemicals may be made by looking at the electronic properties of phytoconstituents (Majeed et al., 2021). The highest occupied and lowest unoccupied molecular orbitals play a crucial role in influencing the reactivity of a chemical species. In general, molecules with higher E_HOMO_ values can donate electrons to low-energy molecules with unfilled molecular orbitals (E_LUMO_). The ability of a molecule to receive electrons may be ascertained by looking at the value of E_LUMO_. A molecule becomes more reactive when the HOMO–LUMO energy gap (δE) shrinks, and the resulting molecule exhibits reduced stability.

Chemical hardness is connected with chemical stability and reactivity. If a molecule has a wide HOMO–LUMO energy gap, it is assumed to be stronger, less reactive, and more stable. The HOMO–LUMO energy gap gets smaller the softer and more reactive a molecule is. Similarly, less stable and more reactive compounds are frequently linked to greater electronic chemical potential. A molecule’s tendency to take electrons is determined by the electrophilicity index (ω). A molecule’s stability declines, and its reactivity rises as its electrophilicity rises. Table 6 displays the outcomes of DFT-based calculations for band energy gap and molecular orbital energy descriptors.

**Table 6:** DFT results of the five Cordifolisides.

Sl No.	Molecule name	Optimized energy (eV)	E_HOMO_	E_LUMO_	Eg	Chemical hardness (η)	Electronegativity (χ)	Chemical potential (μ)	Electrophilicity index (ω)	Chemical softness (Ѕ)
1	Cordifoliside A	−1946.201	−0.241	−0.025	0.216	0.108	0.133	−0.133	0.082	9.258
2	Cordifoliside B	−1946.191	−0.244	−0.050	0.194	0.097	0.147	−0.147	0.112	10.302
3	Cordifoliside C	−1946.192	−0.234	−0.049	**0.185**	**0.092**	0.142	−0.142	0.109	**10.818**
4	Cordifoliside D	−2025.471	−0.242	−0.027	0.214	0.107	0.135	−0.135	0.084	9.328
5	Cordifoliside E	−2025.483	−0.234	−0.028	0.206	0.103	0.131	−0.131	0.083	9.696

The reduced HOMO-LUMO energy gap (0.185), the lower hardness (0.092) and higher softness (10.818) of Cordifoliside C confirms that it is the most reactive compound of the Cordifoliside class.

**Figure 12:**
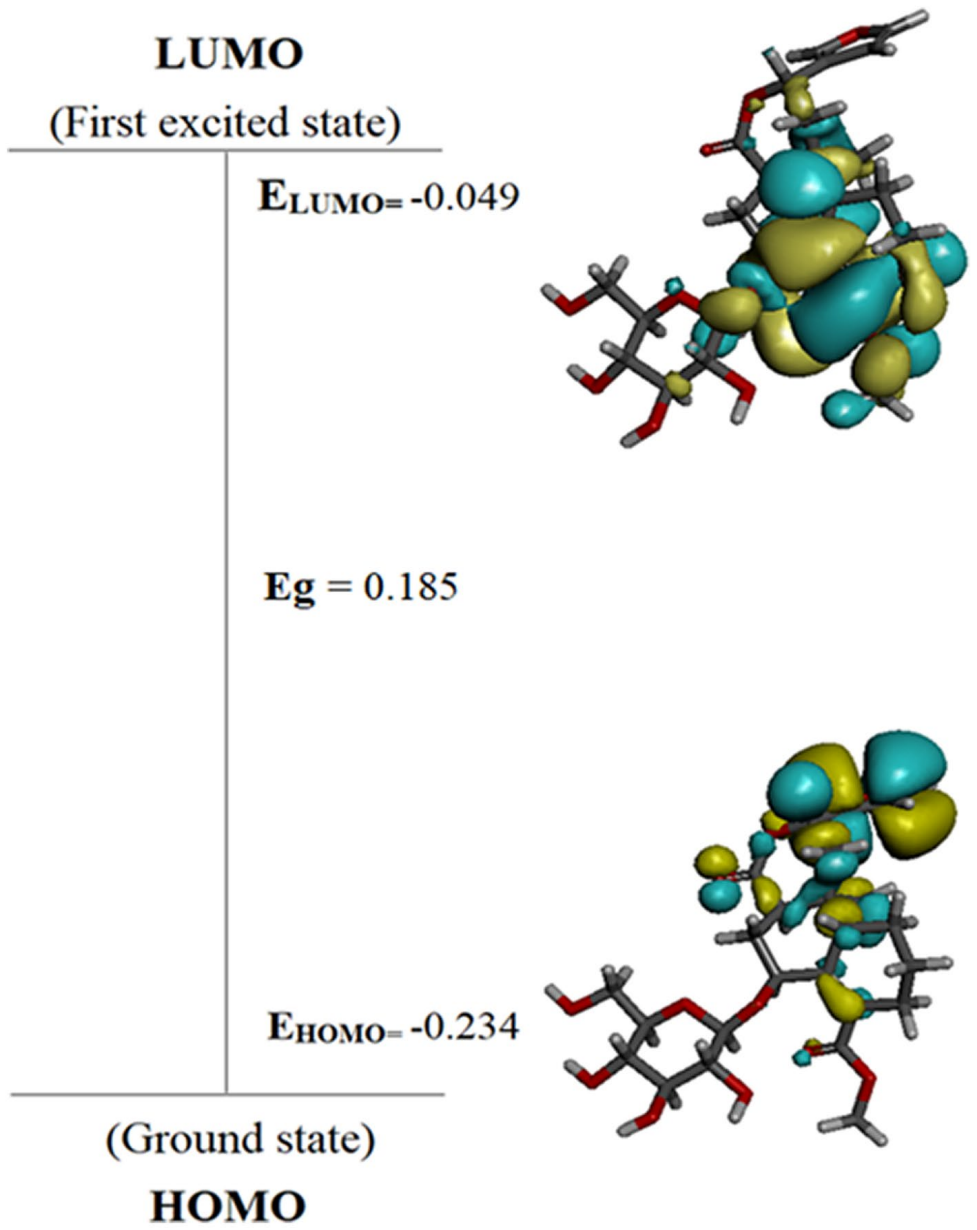
HOMO–LUMO energy diagram of most reactive compound-Cordifoliside C.

Table 6 shows that, among the other compounds used in this investigation, Cordifoliside C displayed the lowest HOMO–LUMO energy gap (Eg). The reduced HOMO–LUMO energy gap (**Eg = 0.185**), caused by the electron acceptor group’s strong capacity to receive electrons, causes charge transfer inside the molecule. Compared to the other molecules, Cordifoliside C also showed lower hardness (0.092) and higher softness (10.818). Figure 12 shows the DFT-optimized geometry and frontier molecular orbitals of Cordifoliside C using B3LYP functional. Therefore, as Cordifoliside C showed the lowest HOMO–LUMO energy gap, with highest softness and lowest hardness values, therefore, Cordifoliside C was chosen for the molecular docking studies.

### Target protein and binding site prediction

As Cordifolisides, together with nanofabricated extracts, showed enhanced antimicrobial activity toward *Pseudomonas aeruginosa*, therefore we tried to understand its mechanism of action against the TolB protein, which is essential for *P. aeruginosa* growth.

**Figure 13:**
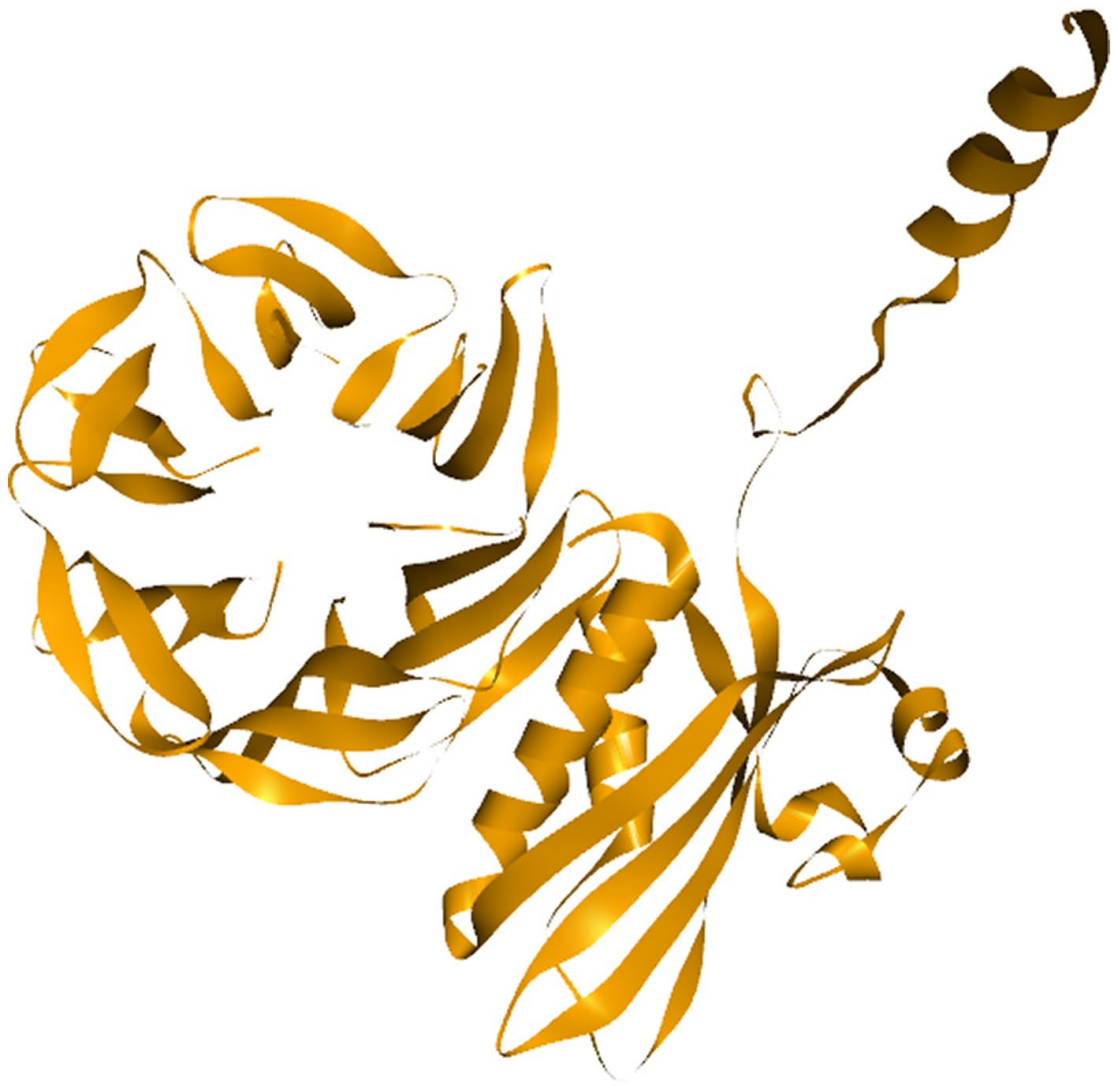
TolB as a target protein.

TolB (Figure 13) is a 44-kDa periplasmic protein partially associated with the outer membrane (Bouveret et al., 1995). It consists of two domains, an N-terminal α/β domain and a C-terminal six-bladed β-propeller (Bonsor et al., 2009). In the TolB/Pal complex(PDBID: 2HQS), Pal binds in the β -propeller domain’s bowl, obstructing the central channel and coming into touch with all of the connecting loops and a number of the β -strands of the blades (Bonsor et al., 2007). Due to direct communication with both membrane-embedded parts, TolA in the inner membrane and Pal in the outer membrane, TolB has a crucial intermediate role in the Tol-Pal system (Bonsor et al., 2009).

The binding region of TolB/Pal complex was predicted using both the CASTp server as well as the “edit binding site” module of DS v4.5.

### Targeted molecular docking studies

**Figure 14:**
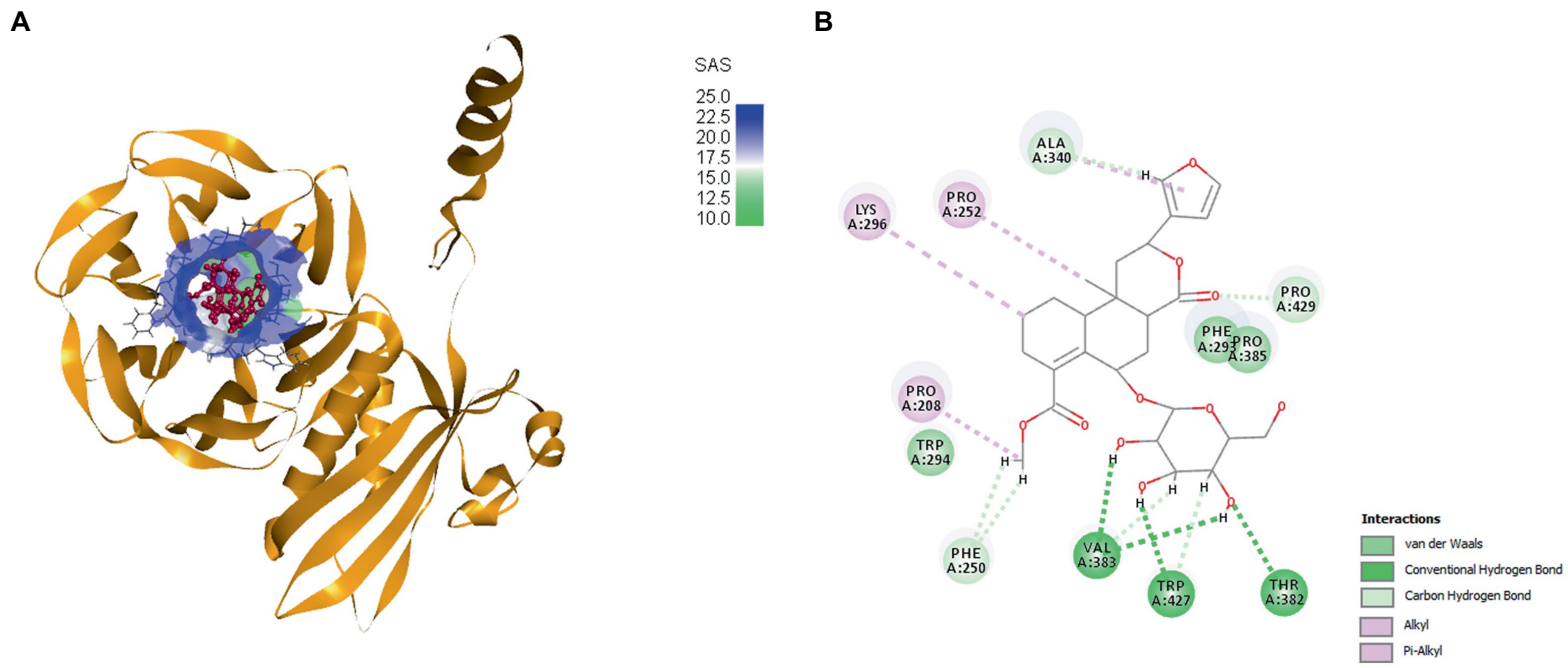
**(A)** Docked pose of Cordifoliside C with TolB, **(B)** Various bonded and non-bonded interactions of Cordifoliside C with TolB.

Molecular docking experiments were done to comprehend and unravel the relationship between the glycoside Cordifoliside C and the TolB. Results of the molecular docking are shown in Figure 14. The CDOCKER binding energy was found to be −119.544 kcal/mol. As illustrated in Figure 14B, docking studies suggest that Cordifoliside C interacts with the β -propeller domain and is stabilized by residues that create a variety of non-covalent interactions, including hydrogen bonds (green), alkyl interactions (pink), and van der Waals interactions (mint). The interacting residues are shown in Table 7.

**Table 7:** Different interactions between binding site residues of Cordifoliside C and TolB protein.

Ligand	Protein	Disease	CDOCKER Binding energy (kcal/mol)	Interacting amino acid residues		
				Conventional H-bonds	Electrostatic (van der Waals interaction)	Alkyl Interactions
Cordifoliside C	TolB	Nosocomial infections	−119.544	VAL 383	PHE 293	PRO 208
				TRP 427	PRO 385	PRO 252
				THR 382	PRO 429	LYS 296

## Discussion

The phytochemical screening of leaves and stem extracts showed the presence of various phytochemicals. It was known from various literature that *T. cordifolia* consists of numerous bioactive compounds. Since most of the compounds were discovered in the methanolic extract, it can be claimed that it has more solubility for *T. cordifolia*’s bioactive components than other solvent extracts.

To determine the antioxidant bioactivity of the crude extracts, one of the most reliable and straightforward spectrophotometric approaches for determining the antioxidant potential of plant extracts is the DPPH radical scavenging test (Farahmandfar et al., 2019). Compounds’ capacity to function as hydrogen donors or free radical scavengers has frequently been tested using the relatively stable DPPH radical. As evident from previously reported studies, the stem methanolic extract showed the best antioxidant activity with the IC_50_ value of 32.6 μg/ml, comparable to the IC_50_ value of the standard Quercetin, i.e., 22.7 μg/ml.

Extracts with antioxidant activity are generally known for their antibacterial and antifungal activities. Overall, the methanolic extract of the stem of *Tinospora cordifolia* exhibited good antimicrobial activity, especially against *Pseudomonas aeruginosa*. Based on the antibacterial activity of the stem methanolic extract, it was selected for further study. After isolating pure isolates through flash chromatography, NMR results revealed that the isolated compound matched the profile of the highly polar Cordifoliside family. This was also substantiated through computational studies where five structures of Cordifolisides were put forward. Among them, Cordifoliside C exhibited the lowest HOMO–LUMO energy gap in the DFT studies as performed above. The smaller the gap, the more reactive a molecule is, thus linked to a greater electronic chemical potential.

Based on the previous studies, Cordifoliside C was selected for targeted molecular docking studies against the TolB protein. This protein is essential for *Pseudomonas aeruginosa* growth and reproduction. TolB-deficient cells exhibit major flaws in their ability to withstand the antibacterial effects. Almost all medicines studied, including those presently used to treat *P. aeruginosa* infections, such as the fluoroquinolone ciprofloxacin, the carbapenem imipenem, and the cephalosporin ceftazidime, exhibited an overall increase in sensitivity in cells expressing low levels of TolB. Additionally, *P. aeruginosa* growth *in vitro* is abolished by TolB deletion, which also significantly lowers persistence and pathogenicity in an animal infection model, as well as resistance to human serum and several antibiotics. This data motivates us to investigate TolB as a potential target protein for *P. aeruginosa* drug development (Sciuto et al., 2014). Docking studies revealed that Cordifoliside C interacts with the TolB protein to inhibit the growth of *P. aeruginosa*. The interaction between the target protein and the Cordifoliside C has been stabilized by various bonded and non-bonded interactions and has a very high CDOCKER binding energy of −119.544 kcal/mol. As previously reported, the HER2 protein also shows good binding affinity toward Cordifoliside C (Rajkhowa et al., 2022a). Thus this phytochemical can shed light on how it binds with different target proteins, aiding researchers in understanding the fundamental mode of action of the drug candidate. Ag and Au nanoparticles are also reported to bind to many proteins, such as serum albumin, hemoglobin, lysozyme, etc., showing good binding affinities (Sen et al., 2011; Aich et al., 2020; Shahabadi et al., 2023).

Ag and Au nanoparticles were generated using the recrystallized fraction isolated from the Stem methanolic crude extract. The nanoparticles also showed exceptional antibacterial activity, with a more excellent zone of inhibitions than the isolate. Thus, the synergistic effect of a combination of Ag and Au with the isolate may provide nanoparticles expressing higher levels of antibacterial activity against the opportunistic pathogen *P. aeruginosa*. This warrants further studies to disclose potential antibiotics that can display superior bioactivity than commercially available ones.

## Conclusion

In this study, experimental and computational studies were done to evaluate the biological importance of *the medicinal plant Tinospora cordifolia.* From our study, we can conclude that even at minimal concentration, *T. cordifolia* stem nanoparticles have excellent antibacterial activity, making them a strong source of antibacterial agent against MDR strains of *P. aeruginosa*. Additionally, the produced nanoparticles strengthen the therapeutic benefits *of T. cordifolia* and improve its therapeutic effectiveness. Cordifoliside C was found to be more reactive out of the five Cordifolisides among the Cordifoliside class that was identified by NMR in this study. In-silico studies showed the binding affinities of Cordifoliside C with TolB, which is crucial for *P. aeruginosa* resistance and growth. In addition to the enhanced antimicrobial activity observed in *P. aeruginosa*, docking studies showed good binding interaction between Cordifoliside C and TolB target protein. The study’s work offers enormous potential for the realm of drug design.

## Data availability statement

The datasets presented in this study can be found in online repositories. The names of the repository/repositories and accession number(s) can be found at: https://doi.org/10.6084/m9.figshare.21830175.

## Author contributions

HN, AK, and SR performed the plant extraction and isolation, anti-microbial as well as the computational studies. KKB performed the nano-functionalization of the isolate. All authors contributed to the article and approved the submitted version.

## Conflict of interest

The authors declare that the research was conducted in the absence of any commercial or financial relationships that could be construed as a potential conflict of interest.

## Publisher’s note

All claims expressed in this article are solely those of the authors and do not necessarily represent those of their affiliated organizations, or those of the publisher, the editors and the reviewers. Any product that may be evaluated in this article, or claim that may be made by its manufacturer, is not guaranteed or endorsed by the publisher.

## Footnotes

^1^ https://charaideo.gov.in/portlets/charaideo-at-a-glance

^2^ https://www.rcsb.org/
